# Lu-177 PSMA vs Comparator Treatments and Survival in Metastatic Castration-Resistant Prostate Cancer

**DOI:** 10.1001/jamanetworkopen.2024.33863

**Published:** 2024-09-17

**Authors:** Yu Yang Soon, Ian C. Marschner, Manjula Schou, Michael S. Hofman, Louise Emmett, Ian D. Davis, Martin R. Stockler, Andrew J. Martin

**Affiliations:** 1 NHMRC Clinical Trials Centre, University of Sydney, Sydney, New South Wales, Australia; 2 Department of Radiation Oncology, National University Cancer Institute, Singapore, Singapore; 3 Department of Medicine, Yong Loo Lin School of Medicine, National University of Singapore, Singapore; 4 Prostate Cancer Theranostics and Imaging Centre of Excellence; Molecular Imaging and Therapeutic Nuclear Medicine, Cancer Imaging, Peter MacCallum Cancer Centre, Melbourne, Victoria, Australia; 5 The Sir Peter MacCallum Department of Oncology, University of Melbourne, Melbourne, Victoria, Australia; 6 Department of Theranostics and Nuclear Medicine, St Vincent’s Hospital, Sydney, New South Wales, Australia; 7 Faculty of Medicine, University of New South Wales, Sydney, New South Wales, Australia; 8 Eastern Health Clinical School, Monash University, Melbourne, Victoria, Australia; 9 Eastern Health, Melbourne, Victoria, Australia; 10 Department of Medical Oncology, Concord Repatriation General Hospital, Sydney, New South Wales, Australia; 11 Department of Medical Oncology, Chris O’Brien Lifehouse, Sydney, New South Wales, Australia; 12 UQ Centre for Clinical Research, University of Queensland, Brisbane, Queensland, Australia

## Abstract

**Importance:**

Observed treatment effects on overall survival (OS) differed substantially in the first 2 randomized clinical trials of lutetium Lu 177 vipivotide tetraxetan (Lu-177) prostate-specific membrane antigen (PSMA) in metastatic castration-resistant prostate cancer.

**Objective:**

To investigate factors associated with the observed difference in treatment effects on OS, including differences in the risk of crossover from randomized treatment after disease progression.

**Design, Setting, and Participants:**

This comparative effectiveness study used individual participant data from 2 randomized clinical trials, TheraP (A Randomised Phase 2 Trial of 177Lu-PSMA617 Theranostic Versus Cabazitaxel in Progressive Metastatic Castration Resistant Prostate Cancer [ANZUP Protocol 1603]) (n = 200), recruited from February 2018 to September 2019 in Australia, and published data from VISION (An International, Prospective, Open Label, Multicenter, Randomized Phase 3 Study of 177Lu-PSMA-617 in the Treatment of Patients With Progressive PSMA-Positive Metastatic Castration-Resistant Prostate Cancer) (n = 831), recruited from June 2018 to October 2019 in North America and Europe. Individual participant data for OS were reconstructed from VISION using the published survival curves. Data were analyzed February 6, 2018, to December 31, 2021, for TheraP and June 4, 2018, to January 27, 2021, for VISION.

**Interventions:**

TheraP randomized participants to receive treatment with Lu-177 PSMA or cabazitaxel. VISION randomized participants to receive treatment with or without Lu-177 PSMA in addition to physicians’ choice of protocol-permitted treatments (PPT; approved hormonal treatments [such as abiraterone and enzalutamide], bisphosphonates, radiotherapy, denosumab, or glucocorticoids), excluding cabazitaxel.

**Main Outcomes and Measures:**

Patient characteristics, treatment protocols, and OS outcomes of the 2 trials were compared. Estimates of the effect on OS from TheraP were adjusted for crossover from randomly assigned treatment using a rank-preserving structural failure time model (RPSFTM) and inverse probability of censoring weights (IPCW) methods.

**Results:**

The 200 participants in TheraP and 831 participants in VISION were similar in age (median [range], 72 [49-86] vs 71 [40-94] years). Improved OS was observed in the comparator treatment group (cabazitaxel) in TheraP compared with VISION (PPT) (hazard ratio [HR], 0.53 [95% CI, 0.39-0.71]). The Lu-177 PSMA treatment groups in TheraP and VISION had similar OS (HR, 0.92 [95% CI, 0.70-1.19]). In TheraP, 20 of 101 participants in the cabazitaxel group crossed over to Lu-177 PSMA, while 32 of 99 participants in the Lu-177 PSMA arm crossed over to cabazitaxel. No statistically significant differences in OS between the Lu-177 PSMA and cabazitaxel groups of TheraP were observed after controlling for crossover to cabazitaxel: RPSFTM HR, 0.97 (95% CI, 0.60-1.58); IPCW HR, 0.92 (95% CI, 0.65-1.32); RPSFTM HR, 0.97 (95% CI, 0.60-1.58) and IPCW HR, 0.82 (95% CI, 0.54-1.24) for crossover to Lu-177 PSMA; RPSFTM HR, 0.96 (95% CI, 0.53-1.74) and IPCW HR, 0.82 (95% CI, 0.53-1.27) for crossover to either Lu-177 PSMA or cabazitaxel.

**Conclusions and Relevance:**

Findings of this secondary analysis of the TheraP and VISION randomized clinical trials suggest that the choice of comparator treatments (ie, cabazitaxel vs PPT) may explain the difference in the observed effect of Lu-177 PSMA on OS between the 2 trials. Causal inference methods such as RPSFTM and IPCW may help rule out crossover as a plausible explanation.

## Key Points

**Question:**

What factors are associated with the difference in the observed effect of treatment with lutetium Lu 177 vipivotide tetraxetan (Lu-177) prostate-specific membrane antigen (PSMA) on overall survival (OS) among patients with metastatic castration-resistant prostate cancer between the TheraP and VISION randomized clinical trials?

**Findings:**

In this comparative effectiveness study using data from 2 randomized clinical trials involving 200 patients in TheraP and 831 patients in VISION, differences in the observed effect on OS was primarily associated with the choice of comparator treatments (cabazitaxel in TheraP vs protocol-permitted treatment [eg, hormonal treatments such as abiraterone and enzalutamide, bisphosphonates or denosumab] without cabazitaxel) in VISION.

**Meaning:**

The observed difference in the effect of Lu-177 PSMA on OS in TheraP vs VISION was associated with the use of an active, life-prolonging comparator in TheraP, suggesting that the choice of comparator matters when designing trials.

## Teaser

This comparative effectiveness research investigates factors associated with the difference in the observed effect of lutetium Lu 177 vipivotide tetraxetan prostate-specific membrane antigen on overall survival between the TheraP and VISION randomized clinical trials among patients with metastatic castration-resistant prostate cancer.

## Introduction

Prostate-specific membrane antigen (PSMA) is a cell surface membrane protein highly expressed on prostate cancer cells.^1,2^ Lutetium Lu 177 vipivotide tetraxetan (Lu-177) PSMA was approved by the US Food and Drug Administration March 23, 2022, for the treatment of PSMA-positive, metastatic castration-resistant prostate cancer (mCRPC) previously treated with an androgen receptor pathway inhibitor and taxane-based chemotherapy.^3^ Lu-177 PSMA, consisting of a beta particle emitter that is linked to a PSMA-binding ligand, selectively binds to PSMA and delivers high doses of radiation to prostate cancer cells, causing their death.^4,5,6,7^

TheraP (A Randomised Phase 2 Trial of 177Lu-PSMA617 Theranostic Versus Cabazitaxel in Progressive Metastatic Castration Resistant Prostate Cancer [ANZUP Protocol 1603]) evaluated treatment with Lu-177 PSMA vs cabazitaxel in patients with PSMA-positive mCRPC that had progressed after chemotherapy with docetaxel.^8^ TheraP demonstrated greater activity for the serum prostate-specific antigen (PSA) response with Lu-177 PSMA compared with cabazitaxel (66% vs 37%) but similar overall survival (OS) outcomes (stratified hazard ratio [HR], 0.97 [95% CI, 0.70-1.35]); *P* = .99) with a median follow-up of 36 [IQR, 31-39] months.^9^

VISION (An International, Prospective, Open Label, Multicenter, Randomized Phase 3 Study of 177Lu-PSMA-617 in the Treatment of Patients With Progressive PSMA-Positive Metastatic Castration-Resistant Prostate Cancer) evaluated treatment with Lu-177 PSMA plus protocol-permitted treatment (PPT) vs PPT alone in patients with PSMA-positive mCRPC that had progressed after 1 or 2 lines of taxane-based chemotherapy and androgen receptor pathway inhibition.^10^ The PPT included approved hormonal treatments (such as abiraterone and enzalutamide), bisphosphonates, radiotherapy, denosumab, or glucocorticoids but excluded cabazitaxel. VISION showed that adding Lu-177 PSMA to the PPT improved imaging-based progression-free survival (PFS) (HR, 0.40 [99.2% CI, 0.29-0.57]) and OS (HR, 0.62 [95% CI, 0.52-0.74]) with a calculated median follow-up of 20 (IQR, 17-23) months.

The HRs for OS observed in TheraP and VISION differed significantly (*P* = .02, Cochran *Q* test for heterogeneity) (eFigure 1 in Supplement 1). Plausible explanations include differences in baseline characteristics, activity of the treatment comparators used (cabazitaxel vs PPT), and the risk of crossover from randomized treatment after disease progression. In TheraP, approximately 20% of participants in the comparator group were subsequently treated with Lu-177 PSMA after disease progression, whereas this occurred in only 0.5% of the VISION comparator group.^8,10^ Approximately 19% of the VISION comparator group were subsequently treated with cabazitaxel. In TheraP, approximately 32% of participants in the intervention group were subsequently treated with cabazitaxel after disease progression, whereas this occurred in 13% of the VISION intervention group.

Treatment crossover can be considered a type of intercurrent event, that is, an event that may obscure the effect of a randomized treatment on an outcome.^11^ The resulting estimation challenge may be addressed using causal inference methods developed initially for observational studies to isolate the association of an exposure variable with an outcome.^12^ This study aimed to evaluate these possible explanations for the different effects on OS observed in TheraP and VISION by using causal inference models and related methods.

## Methods

TheraP (NCT03392428) was a phase 2 randomized clinical trial conducted in 11 centers in Australia comparing treatments with Lu-177 PSMA and cabazitaxel among patients who had PSMA-positive mCRPC that had progressed after docetaxel, recruited from February 2018 to September 2019. The primary end point was PSA response, defined as a reduction of at least 50% from baseline.^8^ VISION (NCT03511664) was a phase 3 randomized clinical trial conducted in 84 centers across North America and Europe comparing treatment with Lu-177 PSMA plus PPT and PPT in patients who had PSMA-positive mCRPC that had progressed after 1 or 2 lines of taxane-based chemotherapy and androgen receptor pathway inhibition, recruited from June 2018 to October 2019. The 2 primary end points were imaging-based PFS and OS (eTables 1 and 2 in Supplement 1).^10^ TheraP received ethics approval at participating sites and all participants provided signed, written, informed consent, including allowing for their data to be used in subsequent analyses. The Peter MacCallum Cancer Centre in Melbourne, Victoria, Australia, provided ethics approval for the analysis of TheraP data. Information from VISION used in the present study was based on published, aggregated data; hence, specific ethics approval was not sought to use data from the VISION trial. Both primary publications of the TheraP and VISION trial adhered to the Consolidated Standards of Reporting Trials (CONSORT) reporting guideline for randomized clinical trials.^13^

For this comparative effectiveness research study, participant selection criteria and the Lu-177 PSMA treatment procedure for TheraP were obtained from the protocol and results publication, and individual participant data from the study database.^8^ Aggregate baseline and patient characteristics information were obtained from the primary publication of the VISION trial.^10^ The individual participant data for OS from VISION were reconstructed from the published Kaplan-Meier curves using WebPlotDigitizer and R package IPDfromKM (R Project for Statistical Computing).^14,15^

We compared the following baseline characteristics of participants in the 2 trials: age, performance status, serum PSA level, serum alkaline phosphatase level, presence of bone metastases, and liver metastases, Gleason score (range 8-10, with higher scores indicating worse prognosis), and previous systemic therapies, including abiraterone, enzalutamide, docetaxel, and cabazitaxel. These characteristics were chosen based on their potential as prognostic or predictive markers for OS or response to Lu-177 PSMA.

Adjustment for crossover in TheraP involved 3 steps. The first step was to construct a detailed specification of the treatment effects we sought to estimate (ie, an estimand). For example, 1 estimand targeted the effect of allocation to Lu-177 PSMA vs allocation to cabazitaxel followed by no crossover after disease progression to Lu-177 PSMA. Second, we explored similarities and differences in baseline characteristics and outcomes between TheraP participants who did or did not cross over. Third, we used rank-preserving structural failure time models (RPSFTM), and inverse probability of censoring weights (IPCW), to estimate the specified estimands.^16,17^ As crossover was negligible (0.5%) in VISION, no adjustment was undertaken.

### Estimand Definition

We used the framework from the International Council for Harmonisation of Technical Requirements for Registration of Pharmaceuticals for Human Use addendum (R1) to E9–Statistical Principles for Clinical Trials framework to specify the following estimands^11^: (1) Lu-177 PSMA with or without crossover vs cabazitaxel with or without crossover; (2) Lu-177 PSMA vs cabazitaxel with no crossover; (3) Lu-177 PSMA with no crossover vs cabazitaxel; and (4) Lu-177 PSMA with no crossover vs cabazitaxel with no crossover (eTable 3 in Supplement 1).

The summary measure for comparisons on OS was the HR calculated from a Cox proportional hazards model. The estimands were constructed for 2 populations of interest: all trial participants and the subgroup of participants with higher PSMA expression levels defined as PSMA mean standard uptake value (SUVmean) greater than or equal to 10.^18^

### RPSFTM

The RPSFTM estimates the outcome of participants who crossed over from their randomized treatment to another treatment under the counterfactual scenario that the crossover never occurred (eMethods 1 in Supplement 1).^16^ This is achieved by amalgamating the survival time accrued during the initial period of adherence to assigned treatment, with the survival time after crossover adjusted by an acceleration factor. The acceleration factor reflects the extent to which survival time is increased or decreased after crossover from randomized treatment. We conducted sensitivity analyses using different models to estimate the acceleration factor, and with prespecified reductions in the estimated acceleration factor.^19^ We used the R package rpsftm, version 4.1.3 (R Project for Statistical Computing), to perform these analyses.^16^

### IPCW

The IPCW calculates and assigns each participant in each treatment group an estimated probability of crossover (eMethods 2 in Supplement 1).^17^ This probability is used to match participants who cross over with participants who do not cross over. Participants who cross over are censored at that time point, and the relative contribution of matched participants who did not cross over is increased by upweighting. The weights used are inversely proportional to the participant’s estimated probability of crossing over. This means that greater weight in the analysis is given to participants who did not cross over and had similar baseline characteristics to participants who did cross over. This adjustment reduces the selection bias due to the censoring of participants who crossed over. We derived weights for the IPCW analysis using logistic regression, with crossover as the binary outcome (yes or no). Covariates investigated included the baseline characteristics of age, hemoglobin level, PSMA SUVmean, volume of ^18^F-fluorodeoxyglucose–avid disease, greater than 20 metastases, study institution, and previous treatment with abiraterone or enzalutamide. The analyses were performed in R (version 4.1.3).^20^

### Statistical Analysis

We used the Cochran *Q* test to determine if the reported OS HR from VISION and the estimated HR from the reconstructed individual time-to-event data from VISION, and the HR from TheraP differed from one another. We used the Kaplan-Meier method to summarize and compare OS between participants assigned to receive Lu-177 PSMA in TheraP vs VISION, and between participants assigned to receive cabazitaxel in TheraP vs PPT in VISION.^8,10^ We used Cox proportional hazards regression to estimate HRs and their 95% CIs. Data were analyzed February 6, 2018, to December 31, 2021, for TheraP and June 4, 2018, to January 27, 2021, for VISION. Analyses were performed with the R package survival, version 4.1.3. A 2-sided *P* < .05 was considered statistically significant.

## Results

### Baseline Characteristics and Lu-177 PSMA Regimens in TheraP and VISION

Participants in TheraP and VISION were similar in age (median [range], 72 [49-86] vs 71 [40-94] years), Eastern Cooperative Oncology Group (ECOG) performance status of 0 to 1 (191 [96%] vs 768 [92%]), bone metastasis (180 [90%] vs 760 [91%]), liver metastasis (9 [5%] vs 101 [12%]), previous receipt of docetaxel (200 [100%] vs 807 [97%]), and Gleason score 8 to 10 (103 [52%] vs 494 [59%]) (Table 1). The median (range) PSA level was slightly higher in TheraP than in VISION (99 [8-6230] vs 76 [0-8995] ng/mL; to convert to micrograms per liter, multiply by 1.0). Differences in prior treatments included cabazitaxel administration (0 in TheraP vs 316 [38%] in VISION) and the number of prior androgen receptor pathway inhibitors used (152 participants [76%] in TheraP vs 426 [51%] in VISION used 1 inhibitor; 30 participants [15%] in TheraP vs 341 [41%] in VISION used 2 inhibitors).

**Table 1.** Baseline Characteristics of Participants in the TheraP and VISION Randomized Clinical Trials

Characteristic	Participants, No. (%)						*P* value, TheraP vs VISION^a^
	TheraP trial			VISION trial			
	Lu-177 PSMA (n = 99)	Cabazitaxel (n = 101)	Overall (n = 200)	Lu-177 PSMA + PPT (n = 551)	PPT alone (n = 280)	Overall (n = 831)	
Age, median (range), y	72 (49-86)	72 (51-84)	72 (49-86)	70 (48-94)	72 (40-89)	71 (40-94)^b^	NA
ECOG performance status 0 or 1	95 (96)	96 (95)	191 (96)	510 (93)	258 (92)	768 (92)	.16
PSA, median (range), ng/mL	94 (8-6230)	110 (15-2730)	99 (8-6230)	78 (0-6988)	75 (0-8995)	76 (0-8995)^b^	NA
ALP, median (range), IU/L	111 (35-1070)	130 (31-1947)	115 (31-1947)	105 (17-2524)	95 (28-1355)	100 (17-2524)^b^	NA
Site of disease, bone	90 (91)	90 (89)	180 (90)	504 (92)	256 (92)	760 (91)	.49
Site of disease, liver	4 (4)	5 (5)	9 (5)	63 (11)	38 (14)	101 (12)	<.001
Gleason score 8-10^c^	53 (53)	50 (50)	103 (52)	324 (59)	170 (61)	494 (59)	.046
Previous treatment with 1 androgen receptor pathway inhibitor^d^	70 (71)	82 (81)	152 (76)	298 (54)	128 (46)	426 (51)	<.001
Previous treatment with 2 androgen receptor pathway inhibitors^d^	21 (21)	9 (9)	30 (15)	213 (39)	128 (46)	341 (41)	<.001
Previous treatment with docetaxel	99 (100)	101 (100)	200 (100)	534 (97)	273 (98)	807 (97)	.008
Previous treatment with cabazitaxel	0	0	0	209 (38)	107 (38)	316 (38)	<.001

Abbreviations: ALP, alkaline phosphatase; ECOG, Eastern Cooperative Oncology Group; Lu-177, lutetium Lu 177 vipivotide tetraxetan; NA, not available; PPT, protocol-permitted treatment; PSA, prostate-specific antigen; PSMA, prostate-specific membrane antigen.

SI conversion factors: To convert ALP levels to microkatals per liter, multiply by 0.0167; PSA levels to micrograms per liter, by 1.0.

^a^ Binary variables were compared with the Fisher exact test. Individual participant data from VISION were unavailable to compare the 2 trials on continuous variables.

^b^ Median was calculated as an average of the median values from the 2 randomized groups.

^c^ Gleason scores range from 8 to 10, with higher scores indicating worse prognosis.

^d^ Androgen receptor pathway inhibitors were defined as enzalutamide and abiraterone in the TheraP trial and as enzalutamide, abiraterone, and apalutamide in the VISION trial.

Both trials administered Lu-177 PSMA once every 6 weeks to a maximum of 6 cycles. TheraP used an initial dose of 8.5 gigabecquerels (GBq; 1 Bq is approximately 2.7 × 10^−11^ curies), reducing by 0.5 GBq each cycle, while VISION used a consistent dose of 7.4 GBq per cycle. The total prescribed dose (TheraP, 43.5 GBq vs VISION, 44.4 GBq) and dose intensity were similar.

### Baseline Characteristics of TheraP Participants Who Did or Did Not Crossover

The baseline characteristics of TheraP participants who did or did not cross over were similar within each randomized treatment group for age (median, 71 [IQR, 67-76] vs 73 [IQR, 67-79] years for Lu-177 PSMA; 71 [IQR, 65-74] vs 72 [IQR, 67-77] years for cabazitaxel), ECOG performance status of 0 to 1 (31 [97%] vs 64 [95%] for Lu-177 PSMA; 18 [90%] vs 78 [97%] for cabazitaxel), PSA level (median, 81 [IQR, 39-146] vs 105 [IQR, 53-224] ng/mL for Lu-177 PSMA; 118 [IQR, 64-289] vs 108 [IQR, 64-245] ng/mL for cabazitaxel), and hemoglobin level (median, 12.5 [IQR, 11.8-13.4] vs 12.4 [IQR, 11.6-13.2] g/dL for Lu-177 PSMA; 12.8 [IQR, 12.3-13.3] vs 12.8 [IQR, 12.1-13.7] g/dL for cabazitaxel; to convert to grams per liter, multiply by 10) (Table 2) as well as for the presence of more than 20 metastases (26 [81%] vs 51 [76%] for Lu-177 PSMA; 16 [80%] vs 63 [78%] for cabazitaxel), PSMA SUVmean (median [IQR], 8 [7-10] vs 9 [7-12] for Lu-177 PSMA; 9 [8-11] vs 8 [7-10] for cabazitaxel), and previous use of abiraterone or enzalutamide (22 [69%] vs 48 [72%] for Lu-177 PSMA; 17 [85%] vs 65 [80%] for cabazitaxel). The baseline characteristics were also similar for the subgroups of participants with PSMA SUVmean ≥10 who did or did not cross over within each randomized treatment group: age (median, 70 [IQR, 67-76] vs 75 [IQR, 66-81] years for Lu-177 PSMA; 70 [IQR, 66-73] vs 72 [IQR, 69-76] years for cabazitaxel), ECOG performance status of 0 to 1 (8 [100%] vs 27 [100%] for Lu-177 PSMA; 6 [74%] vs 21 [96%] for cabazitaxel), PSA level (median, 58 [IQR, 45-85] vs 78 [IQR, 36-167] ng/mL for Lu-177 PSMA; 95 [IQR, 68-126] vs 137 [IQR, 85-215] ng/mL for cabazitaxel), and hemoglobin level (median, 12.7 [IQR, 12.5-13.4] vs 12.4 [IQR, 11.8-13.1] g/dL for Lu-177 PSMA; 13.0 [IQR, 12.7-13.7] vs 12.7 [IQR, 12.0-13.8] g/dL for cabazitaxel) (eTable 5 in Supplement 1) as well as for the presence of more than 20 metastases (7 [88%] vs 20 [74%] for Lu-177 PSMA; 7 [88%] vs 16 [73%] for cabazitaxel), and previous use of abiraterone or enzalutamide (4 [50%] vs 16 [59%] for Lu-177 PSMA; 7 [88%] vs 19 [86%] for cabazitaxel).

**Table 2.** Baseline Characteristics of Participants in TheraP Who Did or Did Not Cross Over

Characteristic	Participants, No. (%)			
	Randomized to Lu-177 PSMA (n = 99)		Randomized to cabazitaxel (n = 101)	
	Crossover to cabazitaxel (n = 32)	No crossover to cabazitaxel (n = 67)	Crossover to Lu-177 PSMA (n = 20)	No crossover to Lu-177 PSMA (n = 81)
Age, median (IQR), y	71 (67-76)	73 (67-79)	71 (65-74)	72 (67-77)
Metastases >20^a^	26 (81)	51 (76)	16 (80)	63 (78)
ECOG performance status				
0	15 (47)	27 (40)	11 (55)	33 (41)
1	16 (50)	37 (55)	7 (35)	45 (56)
2	1 (3)	3 (5)	1 (5)	3 (3)
Missing	0	0	1 (5)	0
PSA, median (IQR), ng/mL	81 (39-146)	105 (53-224)	118 (64-289)	108 (64-245)
Hemoglobin, median (IQR), g/dL	12.5 (11.8-13.4)	12.4 (11.6-13.2)	12.8 (12.3-13.3)	12.8 (12.1-13.7)
PMSA SUVmean, median (IQR)	8 (7-10)	9 (7-12)	9 (8-11)	8 (7-10)
Location of study sites by Australian states				
New South Wales	10 (31)	21 (31)	7 (35)	23 (28)
Victoria	8 (25)	19 (29)	3 (15)	28 (35)
Others	14 (44)	27 (40)	10 (50)	30 (37)
Previous treatment with 1 androgen receptor pathway inhibitor^b^	22 (69)	48 (72)	17 (85)	65 (80)

Abbreviations: ECOG, Eastern Cooperative Oncology Group; Lu-177, lutetium Lu 177 vipivotide tetraxetan; PSA, prostate-specific antigen; PSMA, prostate-specific membrane antigen; SUVmean, mean number of counts from all voxels within the whole body, including all lesions pooled together.

SI conversion factors: To convert hemoglobin level to grams per liter, multiply by 10; PSA level to micrograms per liter, by 1.0.

^a^ Assessed using gallium Ga 68 (^68^Ga-PSMA-11) positron emission tomography–computed tomography by central review.

^b^ Androgen receptor pathway inhibitors were defined as enzalutamide and abiraterone.

### Clinical Outcomes for Participants Who Crossed Over Treatments in TheraP

**Figure 1.**
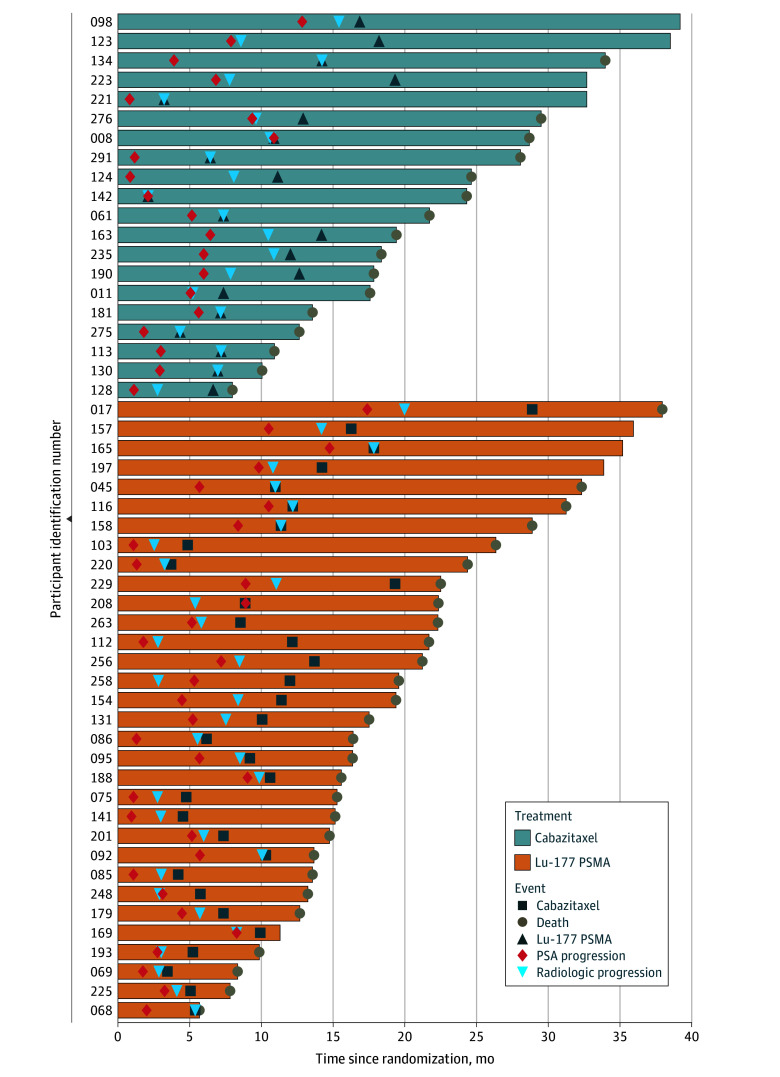
Swimmer Plot of Participants Randomized to Receive Treatment With Cabazitaxel Who Crossed Over to Lutetium Lu 177 Vipivotide Tetraxetan (Lu-177) PSMA Treatment and Participants Randomized To Receive Treatment With Lu-177 PSMA Who Crossed Over to Cabazitaxel Treatment PSA indicates prostate-specific antigen.

All crossovers occurred at or after radiological progression (Figure 1). Among the 101 participants assigned to receive cabazitaxel, 20 crossed over to Lu-177 PSMA, with 16 of them dying before the data cutoff. Among the 99 participants assigned to receive Lu-177 PSMA, 32 crossed over to cabazitaxel, with 28 of them dying before the data cutoff.

### Radiographic PFS (rPFS) and OS by Crossover Status

Nonrandomized comparisons of rPFS and OS for participants who did or did not cross over are summarized in eFigures 2 through 5 in Supplement 1. In the group assigned to receive Lu-177 PSMA, rPFS was shorter in the subgroup that crossed over to cabazitaxel than the subgroup that did not (HR, 2.00 [95% CI, 1.28-3.13]), indicating that participants who crossed over from Lu-177 PSMA to cabazitaxel were likely to have disease progressed earlier than those who did not cross over from Lu-177 PSMA to cabazitaxel (eFigure 2 in Supplement 1). In the group assigned to receive cabazitaxel, rPFS was similar in the subgroups that did or did not cross over to Lu-177 PSMA (HR, 1.23 [95% CI, 0.74-2.04]). In the group assigned to receive Lu-177 PSMA and cabazitaxel, there was limited evidence that OS differed in the subgroups that did or did not cross over (eFigure 3 in Supplement 1). Findings were similar in the subgroup with PSMA SUVmean ≥10 (eFigure 5 in Supplement 1).

### OS Outcomes Adjusted for Crossover

**Figure 2.**
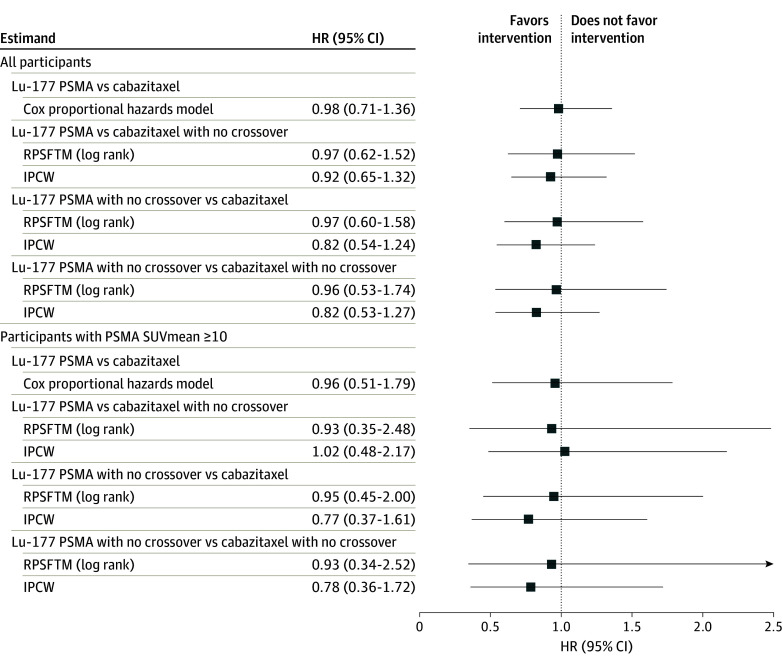
Overall Survival Hazard Ratios for Estimands of Interest HR indicates hazard ratio; IPCW, inverse probability of censoring weights; Lu-177, lutetium Lu 177 vipivotide tetraxetan; PSMA, prostate-specific membrane antigen; RPSFTM, rank-preserving structural failure time model; and SUVmean, mean standard uptake value.

There was no clear evidence that the treatment effect of Lu-177 PSMA vs cabazitaxel on OS differed according to the statistical method and estimands (Figure 2). Among all randomized participants, the unadjusted HR from the conventional Cox proportional hazards model was 0.98 (95% CI, 0.71-1.36). The HR adjusted for crossover using the RPSFTM ranged from 0.96 (95% CI, 0.53-1.74) to 0.97 (95% CI, 0.60-1.58). Conclusions from the RPSFTM were consistent across the various supplementary analyses performed involving different models to estimate the acceleration factor as well as changing the magnitude of the estimated acceleration factor (eTable 4 in Supplement 1). The HR adjusted for crossover using the IPCW ranged from 0.82 (95% CI, 0.53-1.27) to 0.92 (95% CI, 0.65-1.32). Supplementary analyses restricting the numbers of covariates yielded similar HRs (eTable 4 in Supplement 1).

In the subgroup with PSMA SUVmean ≥10, the unadjusted HR from the conventional Cox proportional hazards model was 0.96 (95% CI, 0.51-1.79). The HR adjusted for crossover using the RPSFTM ranged from 0.93 (95% CI, 0.34-2.52) to 0.95 (95% CI, 0.45-2.00). The HR adjusted for crossover using IPCW ranged from 0.77 (95% CI, 0.37-1.61) to 1.02 (95% CI, 0.48-2.17).

### OS in TheraP vs VISION

The analysis of the VISION trial based on the reconstructed data closely matched the published results (HR, 0.62 [95% CI, 0.52-0.74] for the reported VISION results; HR, 0.64 [95% CI, 0.53-0.76] for the reconstructed VISION results) for OS in the Lu-177 PSMA vs PPT groups. Both estimates were different from the estimated HR for TheraP (HR, 0.98 [95% CI, 0.71-1.36]) based on the Cochran *Q* test for heterogeneity (5.89; *df* = 1; *P* = .02).

**Figure 3.**
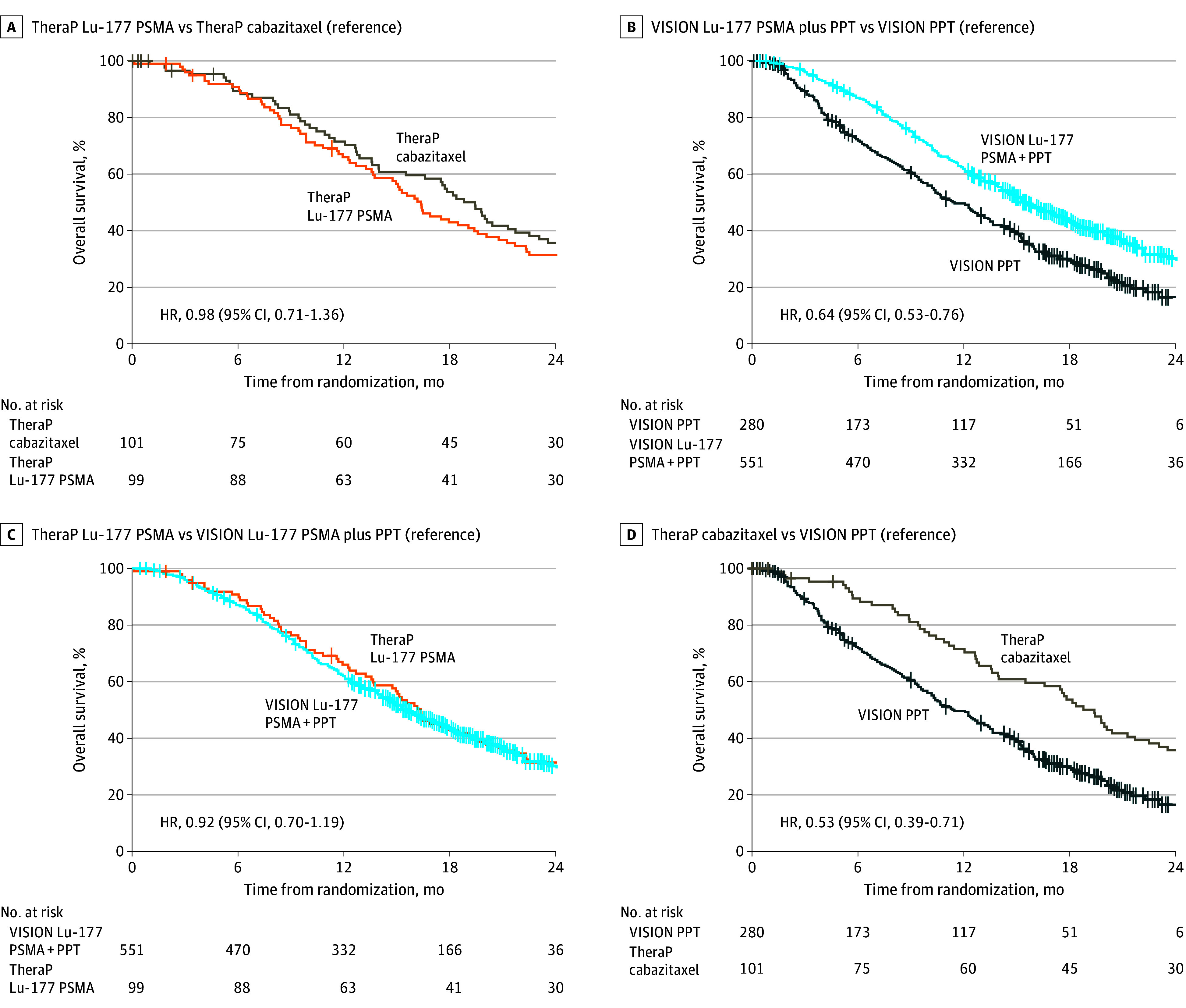
Overall Survival for TheraP and VISION Trials HR represents hazard ratio; Lu-177, lutetium Lu 177 vipivotide tetraxetan; PSMA, prostate-specific membrane antigen; and PPT, protocol-permitted treatment.

The OS was similar among participants assigned to receive Lu-177 PSMA in TheraP vs VISION (HR, 0.92 [95% CI, 0.70-1.19]), with a 24-month survival probability of 31% (95% CI, 23%-42%) for TheraP vs 30% (95% CI, 26%-36%) for VISION (Figure 3). The OS was longer among participants assigned to receive cabazitaxel in TheraP than PPT in VISION (HR, 0.53 [95% CI, 0.39-0.71]), with a 24-month survival probability of 36% (95% CI, 27%-48%) in TheraP vs 17% (11%-24%) in VISION (Figure 3).

## Discussion

This comparative effectiveness research study found little evidence that differences in the estimated effect of Lu-177 PSMA on OS in the TheraP and VISION randomized clinical trials were associated with differences in participant selection, Lu-177 PSMA treatment regimens, or treatment crossover. Statistical adjustments for crossover in TheraP using 2 analytic approaches did not indicate that crossover obscured a true difference in the effects of Lu-177 PSMA vs cabazitaxel on OS.

The comparator treatment chosen for TheraP was cabazitaxel, considered the standard of care for mCRPC that had progressed on or after docetaxel, based on the TROPIC and CARD randomized phase 3 trials.^21,22^ The comparator chosen for VISION was PPT, and chemotherapy with cabazitaxel and other life-prolonging treatments were specifically excluded.^23^

The use of different comparators in the control groups of TheraP and VISION best explained the different treatment effects on OS observed in these trials.^8,10^ Participants assigned Lu-177 PSMA in both trials had remarkably similar survival times, whereas participants assigned PPT in VISION had shorter survival times than those assigned cabazitaxel in TheraP. While cabazitaxel was not a permitted study treatment in VISION, 38% of participants in both intervention and control groups received cabazitaxel prior to randomization. Cabazitaxel was also used after progression in 19% of the VISION control group. In TheraP, cabazitaxel was used by 100% of the control group as the study treatment and by 32% of the Lu-177 PSMA group after progression.

Our findings have several implications. First, Lu-177 PSMA appears to be a promising treatment option to cabazitaxel for mCRPC that has progressed after treatment with androgen receptor signaling inhibitors and docetaxel, and a compelling option where cabazitaxel chemotherapy is considered unsuitable because of prior progression or a contraindication. Second, as we found no association with OS in TheraP after accounting for crossover, any phase 3 comparison of Lu-177 PSMA vs cabazitaxel using OS as the primary end point is likely to be more successful if the trial is designed as a noninferiority trial rather than as a superiority trial. Third, the International Council for Harmonisation estimand framework should be adopted when designing future trials assessing mCRPC. This framework will enable investigators to specify the estimands of interest clearly a priori and to improve the comparability of trials by standardizing the populations, treatments of interest, and methods used to deal with intercurrent events.

### Limitations

This study has limitations. RPSFTM and IPCW are recommended statistical approaches to adjust for crossover in randomized trials^16,17^ but rely on critical assumptions. IPCW assumes that all confounders for crossover have been accounted for and that no unmeasured confounders remain. This assumption remains untestable. We did not include time-varying covariates as possible confounders in the IPCW. RPSFTM assumes that measured and unmeasured confounders are well balanced between groups (which seems highly plausible for a randomized clinical trial such as TheraP). It also assumes that the treatment effect of the crossover agent is the same whether it is given initially at randomization or later after disease progression (the common treatment effect assumption). This assumption is not directly testable, but our findings were consistent across the sensitivity analyses performed, varying the estimated treatment effect in participants who crossed over.

We compared OS times between the Lu-177 PSMA groups in TheraP and VISION, and between the control groups of TheraP and VISION. These comparisons were consistent with the difference in observed treatment effects being associated with differing outcomes in the comparator groups, which may be associated with the comparator regimens themselves or differences in the participant characteristics. More participants in the comparator group of VISION compared with TheraP received 2 androgen receptor pathway inhibitors (46% vs 9%) and cabazitaxel (38% vs 0%). Furthermore, complex analyses that involved covariates could not be performed as the reconstructed individual participant data from VISION did not include this information.^10^ It is possible that other factors, such as differences in the quality of care and access to health care, may have contributed to the differences in the observed treatment effects, but this could not be determined from the published reports of TheraP and VISION.

VISION was designed and powered to detect a clinically important effect on OS, whereas TheraP was not.^8,10^ The smaller sample size of TheraP compared with VISION is not an explanation for the compelling statistical evidence of heterogeneity between trials in HRs for OS.

## Conclusions

The findings of this comparative effectiveness study of 2 randomized clinical trials suggest that the different comparator treatments used in the control groups of TheraP and VISION may be a better supported explanation than differences in treatment crossover or participant characteristics for the observed effects of Lu-177 PSMA on OS. The application of causal inference methods, such as RPSFTM and IPCW, to randomized clinical trial data, is useful for exploring hypotheses about clinically interesting questions (estimands) not directly addressed by the trial.

## Notes

Supplement 1. **eTable 1.** TheraP and VISION trial designs **eTable 2.** Cancer-related therapy after discontinuation of randomized treatment in TheraP and VISION **eTable 3.** Estimands of interest **eTable 4.** Supplementary analysis for overall survival of all participants for the secondary estimands **eTable 5.** Characteristics of participants with PSMA SUVmean ≥10 at baseline for crossovers vs no crossovers, stratified by randomized groups in TheraP **eFigure 1.** Overall survival hazard ratios for VISION and TheraP trials **eFigure 2.** Radiological progression-free survival (rPFS) for crossovers vs no crossovers, stratified by randomized groups for all participants in TheraP **eFigure 3.** Overall survival for crossovers vs no crossovers, stratified by randomized groups for all participants in TheraP **eFigure 4.** Radiological progression-free survival (rPFS) for crossovers vs no crossovers, stratified by randomized groups for participants with PSMA SUVmean ≥10 in TheraP **eFigure 5.** Overall survival for crossovers vs no crossovers, stratified by randomized groups for participants with PSMA SUVmean ≥10 in TheraP **eMethods 1.** Rank preserving structural failure time model **eMethods 2.** Inverse probability of censoring weights

Supplement 2.
Data Sharing Statement
