# Supplementary material for: Lu-177 PSMA vs Comparator Treatments and Survival in Metastatic Castration-Resistant Prostate Cancer
Source: JAMA Netw Open. 2024 Sep 17;7(9):e2433863. doi: 10.1001/jamanetworkopen.2024.33863 (PMC11409154; doi:10.1001/jamanetworkopen.2024.33863)
Supplement: Supplement 1. — eTable 1. TheraP and VISION trial designs eTable 2. Cancer-related therapy after discontinuation of randomized treatment in TheraP and VISION eTable 3. Estimands of interest eTable 4. Supplementary analysis for overall survival of all participants for the secondary estimands eTable 5. Characteristics of participants with PSMA SUVmean ≥10 at baseline for crossovers vs no crossovers, stratified by randomized groups in TheraP eFigure 1. Overall survival hazard ratios for VISION and TheraP trials eFigure 2. Radiological progression-free survival (rPFS) for crossovers vs no crossovers, stratified by randomized groups for all participants in TheraP eFigure 3. Overall survival for crossovers vs no crossovers, stratified by randomized groups for all participants in TheraP eFigure 4. Radiological progression-free survival (rPFS) for crossovers vs no crossovers, stratified by randomized groups for participants with PSMA SUVmean ≥10 in TheraP eFigure 5. Overall survival for crossovers vs no crossovers, stratified by randomized groups for participants with PSMA SUVmean ≥10 in TheraP eMethods 1. Rank preserving structural failure time model eMethods 2. Inverse probability of censoring weights [file jamanetwopen-e2433863-s001.pdf]

Supplementary Online Content

Soon YY, Marschner IC, Schou M, et al. Differential effect of Lu-177 PSMA on survival of metastatic castration-resistant prostate cancer: a secondary analysis of the TheraP and VISION randomized clinical trials. *JAMA Netw Open.* 2024;7(9):e2433863. doi:10.1001/jamanetworkopen.2024.33863

eTable 1. TheraP and VISION trial designs

eTable 2. Cancer-related therapy after discontinuation of randomized treatment in TheraP and VISION

eTable 3. Estimands of interest

eTable 4. Supplementary analysis for overall survival of all participants for the secondary estimands

eTable 5. Characteristics of participants with PSMA SUVmean ≥10 at baseline for crossovers vs no crossovers, stratified by randomized groups in TheraP

eFigure 1. Overall survival hazard ratios for VISION and TheraP trials

eFigure 2. Radiological progression-free survival (rPFS) for crossovers vs no crossovers, stratified by randomized groups for all participants in TheraP

eFigure 3. Overall survival for crossovers vs no crossovers, stratified by randomized groups for all participants in TheraP

eFigure 4. Radiological progression-free survival (rPFS) for crossovers vs no crossovers, stratified by randomized groups for participants with PSMA SUVmean ≥10 in TheraP

eFigure 5. Overall survival for crossovers vs no crossovers, stratified by randomized groups for participants with PSMA SUVmean ≥10 in TheraP

eMethods 1. Rank preserving structural failure time model

eMethods 2. Inverse probability of censoring weights

This supplementary material has been provided by the authors to give readers additional information about their work.

© 2024 Soon YY et al. *JAMA Network Open*.

eTable 1. TheraP and VISION trial designs

Characteristic	TheraP	VISION
**Phase**	Two	Three
**Sponsor**	Academic Cooperative Group	Industry
**Countries involved**	Australia	Belgium, Canada, Denmark, France, Germany, Netherlands, Puerto Rico, Sweden, United Kingdom and United States of America
**Recruitment period**	February 2018 to September 2019	June 2018 to October 2019
**PSMA positive criteria**	^{68}Ga-PSMA-11 maximum standardized uptake value of 20 or greater at a site of disease and greater than 10 for all other measurable metastatic diseases. Sites of disease with discordant 2-[^{18}F] FDG positive and PSMA negative findings were not allowed.	^{68}Ga-PSMA-11 uptake greater than that of liver parenchyma in one or more metastases.
**Previous treatment with Cabazitaxel**	Not allowed	Allowed
**Previous treatment with at least one androgen receptor pathway inhibitor**	Not mandatory	Mandatory
**Treatment for Intervention group**	[^{177}Lu] Lu-PSMA-617, delivered at a starting dose of 8.5GBq and reduced by 0.5 GBq for each subsequent cycle. Each cycle was delivered every six weeks. The maximum number of cycles was six.	Protocol Defined Standard Care + [^{177}Lu] Lu-PSMA-617, delivered 7.4GBq once every six weeks. The maximum number of cycles was six in participants who had evidence of response after four cycles of [^{177}Lu] Lu-PSMA-617.
**Treatment for comparator group**	Cabazitaxel, delivered at 20mg/m^2 once every three weeks. The maximum number of cycles was ten.	Protocol Defined Standard Care (excluded cytotoxic chemotherapies, systemic radioisotopes, immunotherapy)
**Primary endpoint**	PSA response rate defined as reduction of 50% or more from baseline	Imaging progression-free survival<br>Overall survival

© 2024 Soon YY et al. *JAMA Network Open*.

eTable 2. Cancer-related therapy after discontinuation of randomized treatment in TheraP and VISION

	TheraP trial			VISION trial[a]		
**Treatment**	[^{177}Lu] Lu-PSMA-617 (n = 99)	Cabazitaxel (n = 101)	Overall (n = 200)	[^{177}Lu] Lu-PSMA-617 + protocol-permitted treatment (n = 385)	Protocol-permitted treatment alone (n = 196)	Overall (n = 581)
**Abiraterone**	5 (5)	7 (7)	12 (6)	11 (3)	2 (1)	13 (2)
**Cabazitaxel**	32 (32)	21 (21)	53 (27)	51 (13)	38 (19)	89 (15)
**Denosumab**	2 (2)	1 (1)	3 (2)	-	-	-
**Enzalutamide**	2 (2)	9 (9)	11 (6)	6 (2)	4 (2)	10 (2)
**LuPSMA**	5 (5)	20 (20)	25 (13)	0	1 (0.5)	1 (0.2)
**Mitoxantrone**	1 (1)	3 (3)	4 (2)	-	-	-
**Radiotherapy**	13 (13)	15 (15)	28 (14)	25 (7)	22 (11)	47 (8)

[a]Imaging-based progression-free survival analysis set

© 2024 Soon YY et al. *JAMA Network Open*.

eTable 3. Estimands of interest

Estimand	Primary ([^{177}Lu] Lu-PSMA-617 vs Cabazitaxel)	[^{177}Lu] Lu-PSMA-617 vs Cabazitaxel with no crossover to [^{177}Lu] Lu-PSMA-617	[^{177}Lu] Lu-PSMA-617 with no crossover to Cabazitaxel vs Cabazitaxel	[^{177}Lu] Lu-PSMA-617 with no crossover to Cabazitaxel vs Cabazitaxel with no crossover to [^{177}Lu] Lu-PSMA-617
Population	Metastatic Castration-Resistant Prostate Cancer as per trial eligibility criteria			
Variable or endpoint	Overall survival: Time from randomization to death from any cause			
Treatment condition of interest (intervention group)	[^{177}Lu] Lu-PSMA-617 followed by physician choice of subsequent therapies on progression	[^{177}Lu] Lu-PSMA-617 followed by physician choice of subsequent therapies on progression	[^{177}Lu] Lu-PSMA-617 followed by physician choice of subsequent therapies on progression (excluding Cabazitaxel)	[^{177}Lu] Lu-PSMA-617 followed by physician choice of subsequent therapies on progression (excluding Cabazitaxel)
Treatment condition of interest (comparator group)	Cabazitaxel followed by physician choice of subsequent therapies on progression	Cabazitaxel followed by physician choice of subsequent therapies on progression (excluding [^{177}Lu] Lu-PSMA-617)	Cabazitaxel followed by physician choice of subsequent therapies on progression	Cabazitaxel followed by physician choice of subsequent therapies on progression (excluding [^{177}Lu] Lu-PSMA-617)
Strategy for addressing intercurrent events	Treatment policy	Hypothetical		
Population-level summary	Hazard ratio (HR) with confidence interval (CI)			
Estimation	Cox proportional hazards regression model using intention to treat approach	HR from rank preserving structural failure time model using adjusted survival times; adjusted 95% CI was based on p-value from the marginal Cox proportional hazards regression model for the primary estimand.<br><br>Adjusted HR and CI from inverse probability censoring weighted Cox model.		

© 2024 Soon YY et al. *JAMA Network Open*.

eTable 4. Supplementary analysis for overall survival of all participants for the secondary estimands

Secondary Estimand	Model	Hazard ratio	95% CI
**[^{177}Lu] Lu-PSMA-617 vs cabazitaxel with no crossover to [^{177}Lu] Lu-PSMA-617**	RPSFT model (Cox PH)	0.97	0.62 - 1.52
	RPSFT model (Weibull)	0.97	0.60 - 1.56
	RPSFT model (log rank with treatment effect of [^{177}Lu] Lu-PSMA-617 being halved in those who crossover)	0.98	0.68 - 1.41
	IPCW (no quadratic terms for continuous variables)	0.93	0.66 - 1.33
	IPCW (quintiles for continuous variables)	0.95	0.62 - 1.46
	IPCW (performance status and study sites – variables identified based on best subset AIC approach)	1.04	0.73 - 1.48
	IPCW (performance status – variables identified with no zero-coefficient using relax lasso approach with 1SE of lambda)	1.03	0.73 - 1.45
**[^{177}Lu] Lu-PSMA-617 with no crossover to cabazitaxel vs cabazitaxel[a]**	RPSFT model (Cox PH)	0.97	0.60 - 1.58
	RPSFT model (Weibull)	0.96	0.53 – 1.75
	RPSFT model (log rank with treatment effect of [^{177}Lu] Lu-PSMA-617 being halved in those who crossover)	0.98	0.68 - 1.41
	IPCW (no quadratic terms for continuous variables)	0.83	0.55 - 1.24
	IPCW (quintiles for continuous variables)	0.75	0.47 - 1.19
**[^{177}Lu] Lu-PSMA-617 with no crossover to cabazitaxel vs cabazitaxel with no crossover to [^{177}Lu] Lu-PSMA-617[b]**	RPSFT model (Cox PH)	0.96	0.53 - 1.74
	RPSFT model (Weibull)	0.96	0.45 – 2.05
	RPSFT model (log rank with treatment effect of [^{177}Lu] Lu-PSMA-617 being halved in those who crossover)	0.98	0.65 – 1.47

© 2024 Soon YY et al. *JAMA Network Open*.

	IPCW (no quadratic terms for continuous variables)	0.83	0.54 - 1.28
	IPCW (quintiles for continuous variables)	0.74	0.44 - 1.25

PH = Proportional Hazards, RPSFT: Rank Preserving Structural Failure Time, IPCW: Inverse Probability Censoring Weights. [a]No variables were selected using best subset or lasso approach for estimand [^{177}Lu] Lu-PSMA-617 with no crossover to cabazitaxel vs cabazitaxel. [b]IPCW based on best subset or lasso approach was not performed as no variables were selected using these approaches for estimand [^{177}Lu] Lu-PSMA-617 with no crossover to cabazitaxel vs cabazitaxel

© 2024 Soon YY et al. *JAMA Network Open*.

eTable 5. Characteristics of Participants with PSMA SUVmean ≥10 at baseline for crossovers vs no crossovers, stratified by randomized groups in TheraP.

	Randomized to [^{177}Lu] Lu-PSMA-617 (n = 35)		Randomized to Cabazitaxel (n=30)	
	Participants, No. (%)		Participants, No. (%)	
Characteristic	Crossover to Cabazitaxel (n = 8)	No crossover to Cabazitaxel (n = 27)	Crossover to [^{177}Lu] Lu-PSMA-617 (n = 8)	No crossover to [^{177}Lu] Lu-PSMA-617 (n = 22)
Age, median (IQR), years	70 (67-76)	75 (66-81)	70 (66-73)	72 (69-76)
>20 metastases*	7 (88)	20 (74)	7 (88)	16 (73)
ECOG performance status				
0	4 (50)	13 (48)	5 (61)	10 (46)
1	4 (50)	14 (52)	1 (13)	11 (50)
2	0 (0)	0 (0)	1 (13)	1 (4)
Missing	0 (0)	0 (0)	1 (13)	0 (0)
PSA, median (IQR), ng/ml	58 (45-85)	78 (36-167)	95 (68 – 126)	137 (85-215)
Hemoglobin, median (IQR), g/l	127 (125-134)	124 (118 – 131)	130 (127 – 137)	127 (120 – 138)
Location of study sites by states				
New South Wales	5 (63)	10 (37)	3 (38)	6 (28)
Victoria	3 (37)	8 (30)	3 (38)	8 (36)
Others	0 (0)	9 (33)	2 (24)	8 (36)
Previous treatment with one androgen-receptor-pathway inhibitor	4 (50)	16 (59)	7 (88)	19 (86)

© 2024 Soon YY et al. *JAMA Network Open*.

Data are No. (%) or median (IQR). ^{177}Lu = lutetium-177. PSMA = prostate-specific membrane antigen. SUVmean = mean number of counts from all voxels with the whole body, including all lesions pooled together. ECOG = Eastern Cooperative Oncology Group. PSA = prostate-specific antigen. *Assessed using gallium-68[^{68}GA] Ga-PSMA-11 PET-CT by central review. Androgen-receptor-pathway inhibitors were defined as enzalutamide and abiraterone.

© 2024 Soon YY et al. *JAMA Network Open*.

eFigure 1. Overall survival hazard ratios for VISION and TheraP trials

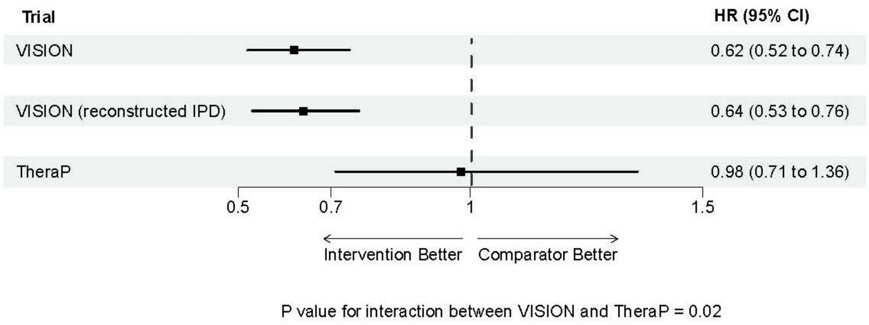

© 2024 Soon YY et al. *JAMA Network Open*.

eFigure 2. Radiological Progression-free Survival (rPFS) for crossovers vs no crossovers, stratified by randomized groups for all participants in TheraP

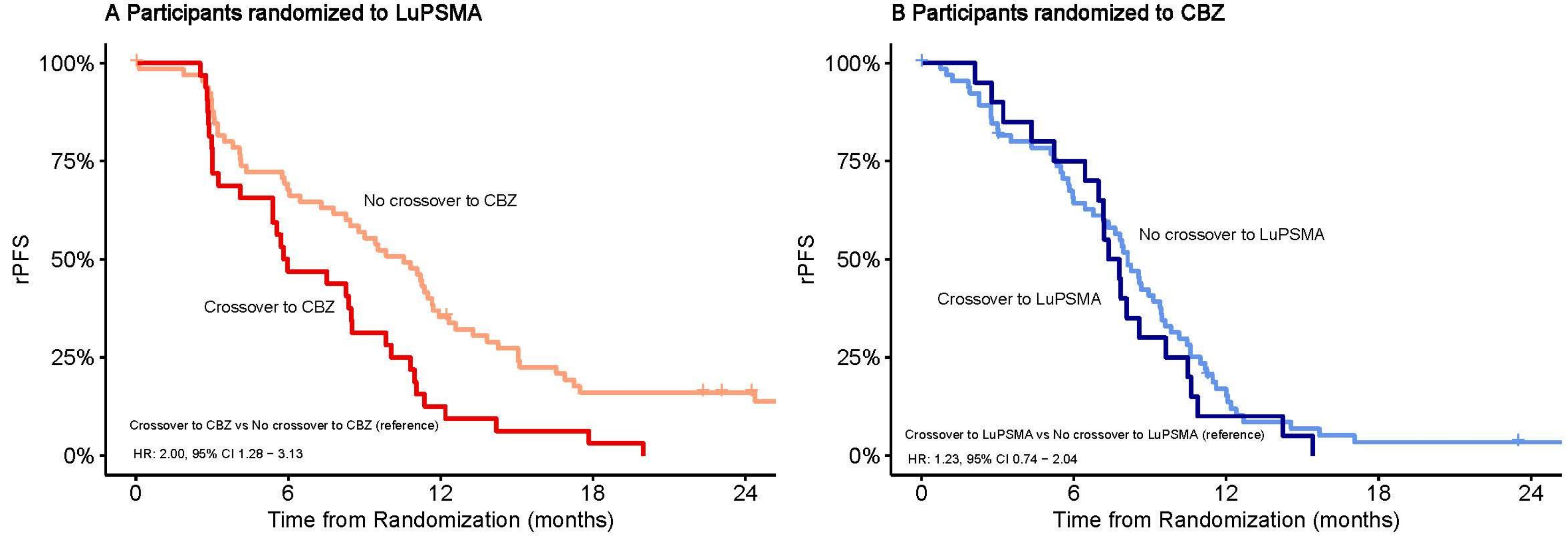

No. at risk

	0	6	12	18	24
No crossover to CBZ	67	44	23	10	8
Crossover to CBZ	32	15	4	1	0

No. at risk

	0	6	12	18	24
No crossover to LuPSMA	81	41	9	2	1
Crossover to LuPSMA	20	15	2	0	0

CBZ= Cabazitaxel, LuPSMA= Lutetium-177-PSMA-617, HR= Hazard ratio, CI= Confidence Interval

© 2024 Soon YY et al. *JAMA Network Open*.

eFigure 3. Overall survival for crossovers vs no crossovers, stratified by randomized groups for all participants in TheraP

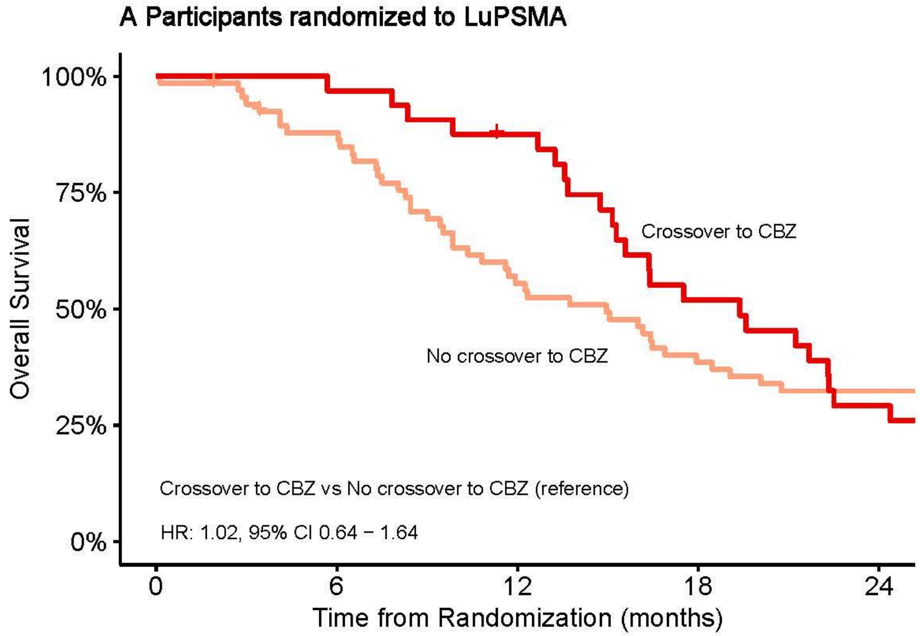

No. at risk

	0	6	12	18	24
No crossover to CBZ	67	57	36	25	21
Crossover to CBZ	32	31	27	16	9

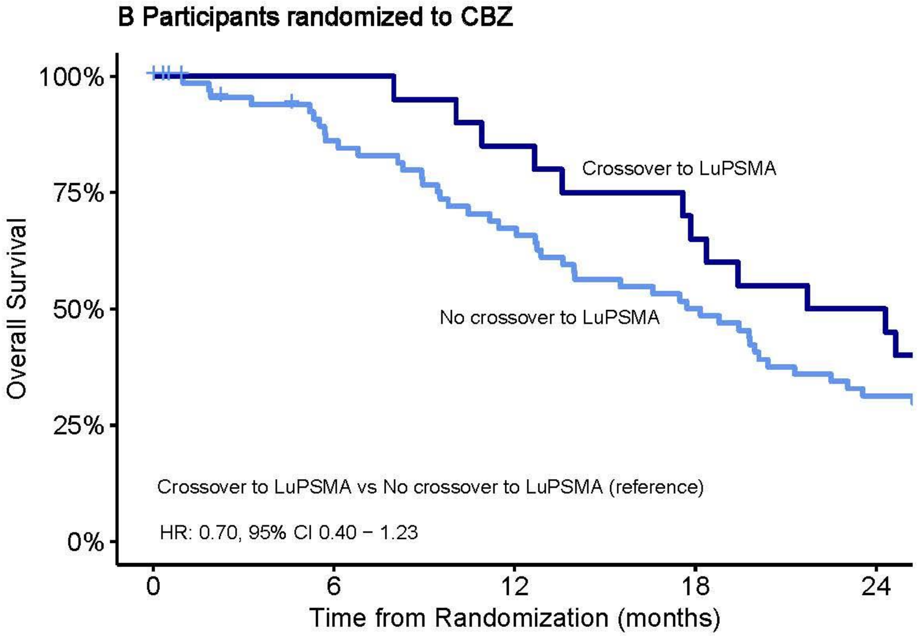

No. at risk

	0	6	12	18	24
No crossover to LuPSMA	81	55	43	32	20
Crossover to LuPSMA	20	20	17	13	10

CBZ= Cabazitaxel, LuPSMA= Lutetium-177-PSMA-617, HR= Hazard ratio, CI= Confidence Interval

© 2024 Soon YY et al. *JAMA Network Open*.

eFigure 4. Radiological progression-free survival (rPFS) for crossovers vs no crossovers, stratified by randomized groups for participants with PSMA SUVmean ≥10 in TheraP

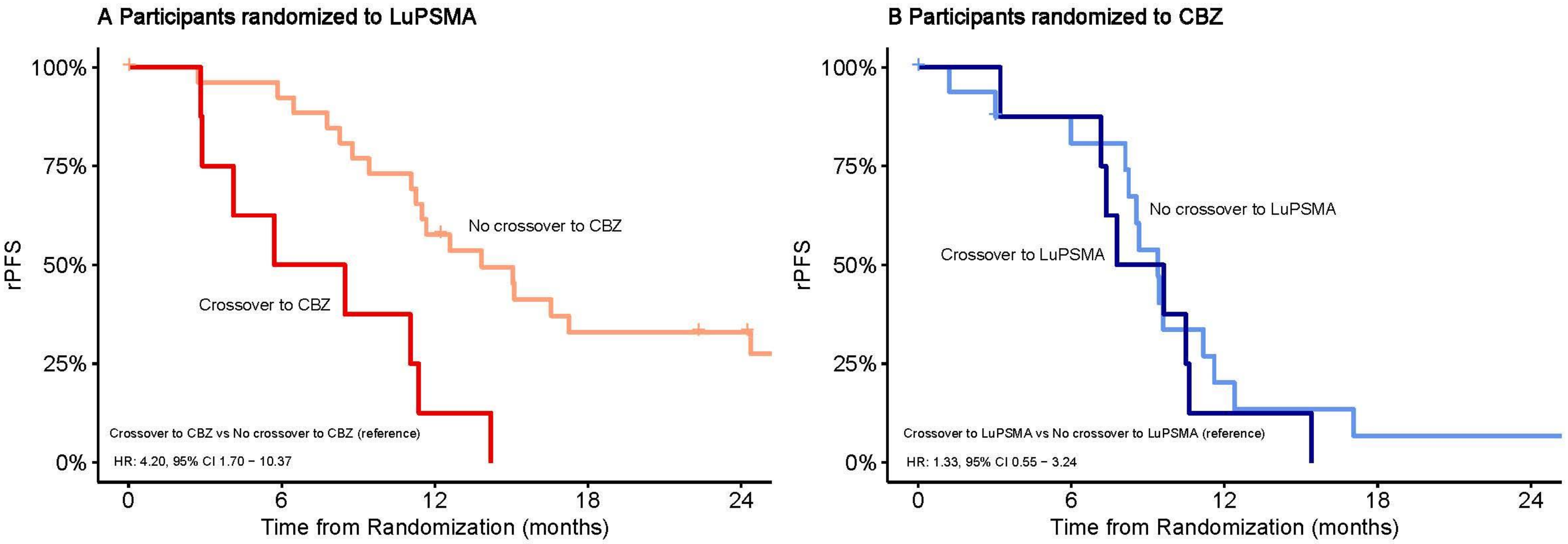

No. at risk

	0	6	12	18	24
No crossover to CBZ	27	24	15	8	7
Crossover to CBZ	8	4	1	0	0

No. at risk

	0	6	12	18	24
No crossover to LuPSMA	22	12	3	1	1
Crossover to LuPSMA	8	7	1	0	0

CBZ= Cabazitaxel, LuPSMA= Lutetium-177-PSMA-617, HR= Hazard ratio, CI= Confidence Interval

© 2024 Soon YY et al. *JAMA Network Open*.

eFigure 5. Overall survival for crossovers vs no crossovers, stratified by randomized groups for participants with PSMA SUVmean ≥10 in TheraP

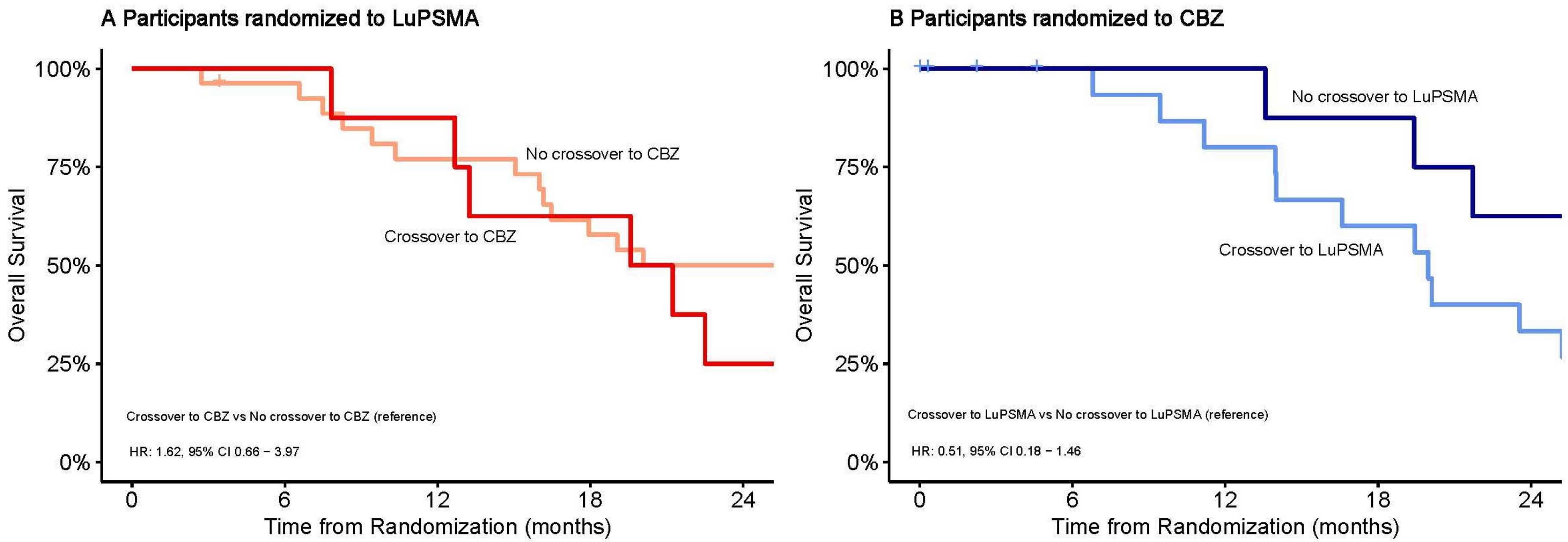

No. at risk

	0	6	12	18	24
No crossover to CBZ	27	25	20	15	13
Crossover to CBZ	8	8	7	5	2

No. at risk

	0	6	12	18	24
No crossover to LuPSMA	22	15	12	9	5
Crossover to LuPSMA	8	8	8	7	5

CBZ= Cabazitaxel, LuPSMA= Lutetium-177-PSMA-617, HR= Hazard ratio, CI= Confidence Interval

© 2024 Soon YY et al. *JAMA Network Open*.

eMethods 1. Rank preserving structural failure time model (RPSFTM)

Main analysis

Acceleration Factor Calculation

The acceleration factor is the extent to which being on the crossover treatment increases or decreases survival and is estimated using an estimation procedure called g-estimation. A value for the acceleration factor is selected from a range of values and input into the counterfactual survival model for all participants to determine the survival time if both randomized groups had received the same assigned treatment and did not have the crossover treatment. The log rank test is used to assess whether the final value of the acceleration factor result in the same average survival times of both groups if they received the same assigned treatment and did not have the crossover treatment. Application of recensoring can used to estimate acceleration factors as well. The aim of recensoring is to reduce the possible bias introduced by censoring times which maybe due to informative censoring when decision to crossover is related to prognostic factors. Recensoring involves shrinking both survival and censoring times of those who had crossover treatment.

Hazard Ratio Calculation

The counterfactual survival times of those who crossover is equal to the time spent on the assigned treatment plus the time spent on the crossover treatment multiplied by the acceleration factor. We used the Cox proportional hazards regression model to estimate the hazard ratio for the secondary estimands of interest based on the observed survival times for those who did not crossover and the counterfactual survival times for those who crossover. The 95% CI was derived using the p value obtained from the intention to treat analysis for the primary estimand.

Supplementary analysis

The Cox proportional hazards model and the Weibull model, instead of the log rank test were used to assess for the final value of acceleration factor as part of the supplementary analysis. To test the

© 2024 Soon YY et al. *JAMA Network Open*.

common treatment effect assumption, we applied a 50% reduction to the treatment effect in participants who had crossover treatment at disease progression compared with the treatment effect where participants received the crossover treatment as their assigned treatment at the start of randomization.

© 2024 Soon YY et al. *JAMA Network Open*.

eMethods 2. Inverse probability of censoring weights (IPCW)

Main analysis

Stabilized weights Calculation

The following covariates (participant's characteristics and disease characteristics at baseline) were selected for the logistic regression to predict for crossover: age, age^2, hemoglobin, $hemoglobin^2$, PSMA SUVmean, PSMA $SUVmean^2$, volume of FDG avid disease, volume of FDG avid $disease^2$, prostate-specific antigen, prostate-specific $antigen^2$, performance status, presence of greater than 20 sites of disease, study site and previous use of abiraterone or enzalutamide.

The conditional probability of crossover from the logistic model forms the denominator of the stabilized weights

The numerator of the stabilized weights is the probability of crossover regardless of any covariates.

The stabilized weights were preferred to the unstabilized weights as stabilized weights give rise to narrower 95% CIs.

Hazard Ratio Calculation

We incorporated the stabilized weights in a Cox proportional hazards regression model with robust variance estimation, adjusted for the covariates used in stabilized weights calculation to estimate the HR and their 95% CIs.

Supplementary analysis

We performed the following supplementary analyses by adjusting the covariates in the logistic regression model. These adjustments involved

a. excluding the quadratic terms of continuous covariates

© 2024 Soon YY et al. *JAMA Network Open*.

b. changing the covariates to quintiles
c. Using the best subset selection based on Akaike Information Criterion (AIC) to identify a subset of variables that accurately predict the outcome
d. Using the relax Lasso regression to select for covariates with non-zero coefficients when the binomial deviance is at the minimum.

Supplementary analysis for participants with PSMA SUVmean ≥ 10

The main analyses of RPSFTM and IPCW method were applied to this subgroup of participants. The covariates selected the logistic regression model were age, age^2, hemoglobin, $hemoglobin^2$, performance status, presence of greater than 20 sites of disease, study site and previous use of abiraterone or enzalutamide. We did not select volume of FDG disease and prostate-specific antigen levels as these covariates would result in extreme probabilities of 0 or 1 for the logistic regression model. We did not use PSMA SUVmean covariate as well since this subgroup of population has PSMA SUVmean of 10 or greater.

© 2024 Soon YY et al. *JAMA Network Open*.
