# Supplementary material for: Lu-177 PSMA vs Comparator Treatments and Survival in Metastatic Castration-Resistant Prostate Cancer
Source: JAMA Netw Open. 2024 Sep 17;7(9):e2433863. doi: 10.1001/jamanetworkopen.2024.33863 (PMC11409154; doi:10.1001/jamanetworkopen.2024.33863)
Supplement: Supplement 2. — Data Sharing Statement [file jamanetwopen-e2433863-s002.pdf]

Data Sharing Statement

Soon. Lu-177 PSMA vs Comparator Treatments and Survival in Metastatic Castration-Resistant Prostate Cancer. *JAMA Netw Open*. Published September 17, 2024. doi:10.1001/jamanetworkopen.2024.33863

Data
Data available: No

Additional Information
Explanation for why data not available: De-identified participant data will be made available to bona fide researchers registered with an appropriate institution following publication. Methodologically sound proposals for any purpose will be considered by the trial executive committee who will have the right to review and comment on any draft manuscripts before publication. Proposals should be directed to michael.hofman@petermac.org. To gain access, data requesters will sign a data access agreement.
